# Identifying effective diagnostic biomarkers and immune infiltration features in chronic kidney disease by bioinformatics and validation

**DOI:** 10.3389/fphar.2022.1069810

**Published:** 2022-12-30

**Authors:** Tao Liu, Xing Xing Zhuang, Xiu Juan Qin, Liang Bing Wei, Jia Rong Gao

**Affiliations:** ^1^ Department of Pharmacy, The First Affiliated Hospital of Anhui University of Chinese Medicine, Hefei, China; ^2^ College of Pharmacy, Anhui University of Chinese Medicine, Hefei, China; ^3^ Department of Pharmacy, Chaohu Hospital of Anhui Medical University, Chaohu, China; ^4^ Anhui Province Key Laboratory of Chinese Medicinal Formula, Hefei, China

**Keywords:** chronic kidney disease, diagnose biomarker, immune cell infiltration, bioinformatics analysis, molecular docking

## Abstract

**Background:** Chronic kidney disease (CKD), characterized by sustained inflammation and immune dysfunction, is highly prevalent and can eventually progress to end-stage kidney disease. However, there is still a lack of effective and reliable diagnostic markers and therapeutic targets for CKD.

**Methods:** First, we merged data from GEO microarrays (GSE104948 and GSE116626) to identify differentially expressed genes (DEGs) in CKD and healthy patient samples. Then, we conducted GO, KEGG, HPO, and WGCNA analyses to explore potential functions of DEGs and select clinically significant modules. Moreover, STRING was used to analyse protein-protein interactions. CytoHubba and MCODE algorithms in the cytoscape plug-in were performed to screen hub genes in the network. We then determined the diagnostic significance of the obtained hub genes by ROC and two validation datasets. Meanwhile, the expression level of the biomarkers was verified by IHC. Furthermore, we examined immunological cells’ relationships with hub genes. Finally, GSEA was conducted to determine the biological functions that biomarkers are significantly enriched. STITCH and AutoDock Vina were used to predict and validate drug–gene interactions.

**Results:** A total of 657 DEGs were screened and functional analysis emphasizes their important role in inflammatory responses and immunomodulation in CKD. Through WGCNA, the interaction network, ROC curves, and validation set, four hub genes (IL10RA, CD45, CTSS, and C1QA) were identified. Furthermore, IHC of CKD patients confirmed the results above. Immune infiltration analysis indicated that CKD had a significant increase in monocytes, M0 macrophages, and M1 macrophages but a decrease in regulatory T cells, activated dendritic cells, and so on. Moreover, four hub genes were statistically correlated with them. Further analysis exhibited that IL10RA, which obtained the highest expression level in hub genes, was involved in abnormalities in various immune cells and regulated a large number of immune system responses and inflammation-related pathways. In addition, the drug–gene interaction network contained four potential therapeutic drugs targeting IL10RA, and molecular docking might make this relationship viable.

**Conclusion:** IL10RA and its related hub molecules might play a key role in the development of CKD and could be potential biomarkers in CKD.

## 1 Introduction

CKD affects approximately 10% of the global population and is mainly characterized by impaired renal functions with persistent inflammation and renal immune response (Lees et al., 2019; Holle et al., 2022). CKD is a public health disease of concern as it can progress to end-stage renal disease (ESKD) that requires dialysis or kidney transplantation (Quon et al., 2011; Jankowski et al., 2021). The exact mechanism of CKD progression is currently unclear, and limited and non-specific treatments remain used to alleviate CKD progression (Harari-Steinberg et al., 2013). Therefore, revealing the pathological mechanisms and exploring the diagnostic biomarkers of CKD are the focus of current research and are the keys to the early diagnosis and treatment of CKD.

In the investigation of the CKD pathogenesis, it has been found that immune responses and inflammatory mediators play significant roles in the condition. Pro-inflammatory factors often reflect elevated inflammatory levels in CKD and ESKD, which leads to a significantly higher mortality rate (Zimmermann et al., 1999; Stenvinkel et al., 2005; Honda et al., 2006; Zoccali et al., 2006; Snaedal et al., 2009; Dekker et al., 2017). Underlying diseases, lifestyle habits, and aging are adverse factors that increase inflammation in CKD (Franceschi et al., 2007; GBD 2015 Mortality and Causes of Death Collaborators, 2016; GBD 2016 Causes of Death Collaborators, 2017). Inflammation may be promoted and maintained by a decreased glomerular filtration rate, reduced cytokine elimination, and metabolic acidosis (Glorieux et al., 2004a; Glorieux et al., 2004b; Platten et al., 2009; Aveles et al., 2010; Ori et al., 2013). When inflammation persists, it can generate organized structures with T cells and lymphatic vessels that correspond to what is called a tertiary lymphoid structure (TLS) (Ruddle, 2014; Sato et al., 2016). TLS has been reported to be associated with a variety of autoimmune kidney diseases, including ANCA-associated glomerulonephritis, systemic lupus erythematosus, membranous glomeruli, and IgA nephritis (Cohen et al., 2005; Segerer and Schlöndorff, 2008; Pei et al., 2014; Seleznik et al., 2016; Brix et al., 2018). B lymphocytes can directly invade non-lymphoid organs such as the kidneys. The chemokine CXCL13 (also known as the B1 cell attractor) expressed in the local stroma could recruit B cells (Legler et al., 1998). These in turn secrete lymphotoxins that promote the differentiation of the perivascular matrix into lymphoid tissue into fibroreticular cells and dendritic cells to consolidate new lymphocyte-like structure (Kratz et al., 1996; Lee et al., 2006; Krautler et al., 2012; Dubey et al., 2017). In human and mouse models, complex B-cell infiltration also occurs in allogeneic immunity, i.e., renal transplant rejection (Steinmetz et al., 2007; Cippà et al., 2019; Kreimann et al., 2020; Steines et al., 2020). Additionally, a number of other immune cells are also key regulators of CKD pathogenesis, such as macrophages and CD4 positive T cells (Rabb et al., 2000; Glassock et al., 2015). However, the immunological mechanism of CKD has not been fully studied. Therefore, evaluating immune cell contributions and exploring key genes associated with immune cells requires a systematic approach, which is an urgent priority.

**FIGURE 1:**
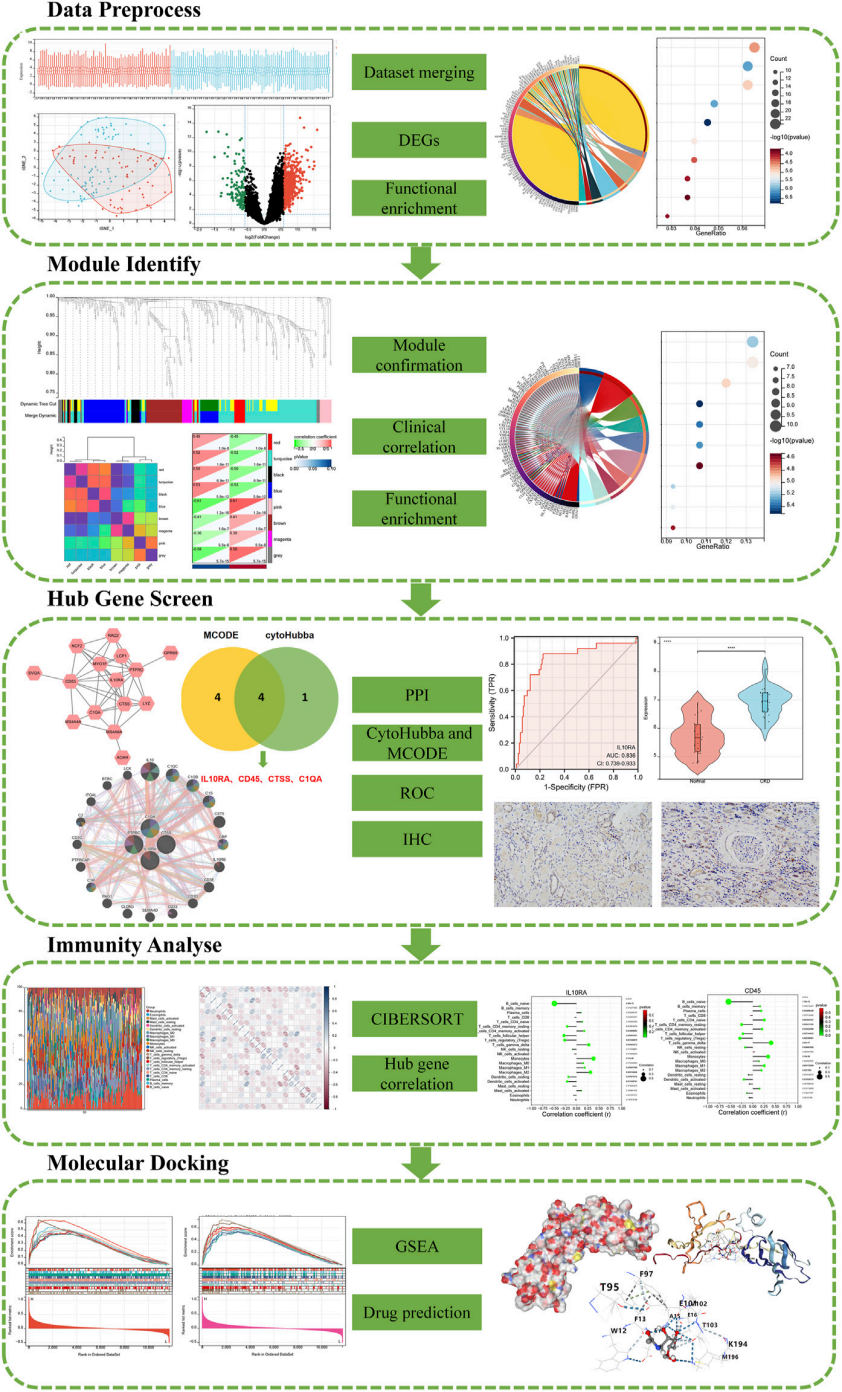
A schematic diagram based on a comprehensive method of bioinformatics analysis and validation experiment of CKD.

In this paper, we conducted a statistical analysis of differential mRNA expression utilizing R tools and the LIMMA package, integrating multiple datasets. A gene weighted co-expression network was constructed according to calculated module associations, gene significance correlations, and inter-module correlations utilizing the R package WGCNA, and DEGs were functionally analyzed with major module genes. STRING was used to study protein interactions between key modular products. Using Cytoscape’s MCODE and MCC algorithms, four hub genes were identified in the network. Furthermore, we used ROC analysis and two datasets to validate the selected signature genes and calculated the relationship between immunity and signature genes by CIBERSORT. For further screening, IL10RA was selected and validated by GSEA analysis, which suggested that IL10RA is strongly involved in various immune and inflammatory responses. Finally, the corresponding therapeutic drugs of IL10RA were predicted and verified by molecule docking. Figure 1 shows the flow chart of our study.

## 2 Materials and methods

### 2.1 Data download and preprocessing

Public microarray data containing clinical information on CKD and normal kidney tissues was obtained from the NCBI GEO GSE104948 and GSE116626 datasets. The GSE104948 data set (RNA was extracted from the glomerular compartment), including 50 CKD kidney samples and 18 normal samples, was based on the Affymetrix Human Genome U133 Plus 2.0 Array of the GPL22945 platform. The GSE116626 data set (RNA was extracted from archival formalin-fixed paraffin-embedded kidney biopsy samples), including 74 CKD kidney samples and 7 normal samples, was based on the Illumina HumanHT-12 WG-DASL V4.0 R2 expression beadchip of the GPL14951 platform. To merge the multiple datasets, we utilized the inSilicoMerging (Taminau et al., 2012) R package to process the datasets. In addition, we used the Johnson et al. method (Johnson et al., 2007) to remove group effects. In total, 124 CKD samples and 25 normal samples of tissues were included in the follow-up analysis of this study.

### 2.2 Differentially expressed genes (DEGs) screening and functional correlation analysis

Here, differential analysis was conducted utilizing the limma R package (Ritchie et al., 2015) to get genes that differ between the CKD group and the control group. The statistical criterion for screening RNA expression was | fold-change (FC) | > 1.5 and *p*-value <.05. Based on the org. Hs.eg.db R package, the KEGG rest API, and the Molecular Signatures Database, we obtained gene annotations for GO, KEGG, and C5, respectively. Then, we performed function analysis utilizing the ClusterProfiler R package to get the DEGs enrichment results. *p*-value <.05 was statistically significant. The maximum gene set is 5000 and the minimum gene set is 5.

### 2.3 Identification of clinically significant modules based on weight gene correlation network analysis (WGCNA)

Using gene expression profiles, we calculated the mean absolute deviation (MAD) for each gene and excluded the 50% of DEGs with the lowest mean absolute deviation. In addition, we used the R package WGCNA to remove outlier DEGs and probes to construct a scale-free co-expression network. Specifically, first the Pearson’s correlation matrices and average linkage method were both performed for all pair-wise Genes. Then, a weighted adjacency matrix was constructed using a power function A_mn = |C_mn|^β (C_mn = Pearson’s correlation between Gene_m and Gene_n; A_mn = adjacency between Gene m and Gene n). β was a soft-thresholding parameter that could emphasize strong correlations between Genes and penalize weak correlations. After choosing the power of 6, the adjacency was transformed into a topological overlap matrix (TOM), which could measure the network connectivity of a Gene defined as the sum of its adjacency with all other Genes for network Gene ration, and the corresponding dissimilarity (1-TOM) was calculated. To classify Genes with similar expression profiles into Gene modules, average linkage hierarchical clustering was conducted according to the TOM-based dissimilarity measure with a minimum size (Gene group) of 10 for the Genes dendrogram. To further analyze the module, we calculated the dissimilarity of module eigen Genes, chose a cut line for module dendrogram, and merged some modules. A total of eight co-expression modules were obtained by merging the modules with a distance less than 0.25. Lastly, GS and MM were calculated according to correlations between gene expressions with clinical subtype and correlations between gene expression and module feature vector, respectively. In the clinically significant module, 16 highly connective genes were screened as key genes according to the cut-off criteria [(MM) > 0.8 and (GS) > 0.1].

### 2.4 Protein–protein interaction (PPI) network and hub gene analyses

The PPI networks for modules with very robust filtering conditions (score >0.7) were analyzed using the STRING database. Cytoscape Software (version 3.8.2) was utilized to visualized these PPI networks. The main functional modules were analyzed using Cytoscape’s Molecular Complex Detection Technology (MCODE) plug-in. Selection criteria are defined as follows: K Core = 2, Cut Grade = 2, Maximum Depth = 100, Cut Node Score = 0.3. Cytoscape’s plugin cytoHubba uses the MCC (Maximum Clique Centrality) algorithm to score each node gene. The pivot genes were screened using the top 5 nodal genes of each algorithm’s MCC score. Predictions of gene function and mapping genes with comparable effects were generated by GeneMANIA, a website for constructing PPI networks. Some of the bioinformatics methods employed by network integration algorithms are physical interactions, co-expression, co-localization, gene enrichment analysis, genetic interactions, and locus prediction. In this study, we used GeneMANIA to identify PPI networks of eigengenes.

### 2.5 Diagnostic value of characteristic biomarkers and data validation in CKD

To verify the predicted value of the screende hub genes, we constructed a logistics model using PROC in the R package (version 3.6.3) and used the GGPLOT2 package to visualize the results. The diagnostic value of the identified biomarkers was assessed by the area under the ROC curve (AUC, AUC was between 0.5 and 1). The closer the AUC is to 1, the more effective the diagnosis is. In addition, we performed a controlled reliability analysis using the RNA expression datasets GSE93798 and GSE104066 as validation sets. GSE93798 includes 20 CKD samples and 22 control samples (RNA was extracted from the glomerular compartment). GSE104066 includes 70 CKD samples and 6 control samples (RNA was extracted from the glomerular compartment).

### 2.6 Immunohistochemistry (IHC)

Paraffin-embedded kidney tissue sections from Chaohu Hospital of Anhui Medical University, including CKD patient group (n = 3) and normal control group (n = 3), were obtained according to Institutional Review Board-approved protocols, and informed consent forms were signed by the patients. The expression of IL10RA (1:50, ZENBIO, China), CD45 (1:50, ZENBIO, China), CTSS (1:50, ZENBIO, China), and C1QA (1:50, Affinity, USA) were detected according to the instructions of the immunohistochemistry kit (ZSBIO, China).

### 2.7 Evaluation of immune cell infiltration and correlation analysis between diagnostic markers and infiltrating immune cells

CIBERSORT (Chen et al., 2018) transforms normalized gene expression matrices into immune cell invasiveness components to estimate the relative frequency of immune invasion and is a 1,000 permutation deconvolution algorithm. Then build a histogram to display the 22 types of content. A correlation heatmap of immune cell infiltration in each sample was developed to visualize correlations between immune cell subtypes. Additionally, differential analysis between CKD and normal tissue immune cells was also visualized by a violin plot. Importantly, associations between the identified biomarkers and the level of infiltrating immune cells were explored and visualized by dot-bar graphs using Spearman’s rank correlation analysis.

### 2.8 Gene set enrichment analysis (GSEA) of IL10RA

We utilized the GSEA analysis (Subramanian et al., 2005) to explore regulatory target genes, biological process (BP) GO terms, KEGG pathways, and Human phenotype Ontology in which the selected IL10RA might be involved in CKD. The samples were divided into low expression group (<50%) and high expression group (≥50%) according to the expression level of IL10RA. Datasets in the Molecular Signatures Database, including c3.all.v7.4. symbols.gmt, c5. go.bp.v7.4.symbols.gmt, c2.cp.kegg.v7.4.symbols.gmt, and c5.hpo.v7.4.symbols.gmt, were served as reference gene sets. Statistical significance was assessed by comparing the enrichment score with the enrichment results generated from 1000 random permutations of the gene set to obtain *p*-value, and *p* < .05 was considered significant for GSEA analysis using default parameters.

### 2.9 Drug-gene interaction and molecular docking analyses of IL10RA

To explore drug-gene interactions, existing or/and potentially relevant drug substances were identified using the STITCH database (Szklarczyk et al., 2016). The PubChem database (Kim et al., 2021) and the PDB database (Karuppasamy et al., 2020) were used to obtain the molecular structures of ligands and target proteins. Docking simulations and visualization were performed through PyMOL software (Nguyen et al., 2020) and AutoDock Vina (Lam and Siu, 2017).

### 2.10 Statistical analysis

All data were processed and analyzed using R software. The Mann-Whitney *U* test (Wilcoxon rank sum test) was used to analyze differences between two groups of continuous non-normal variables. A possible correlation between two variables was detected by the Pearson correlation coefficient. *p* < .05 considered the difference to be statistically significant.

## 3 Results

### 3.1 Data preprocessing

**FIGURE 2:**
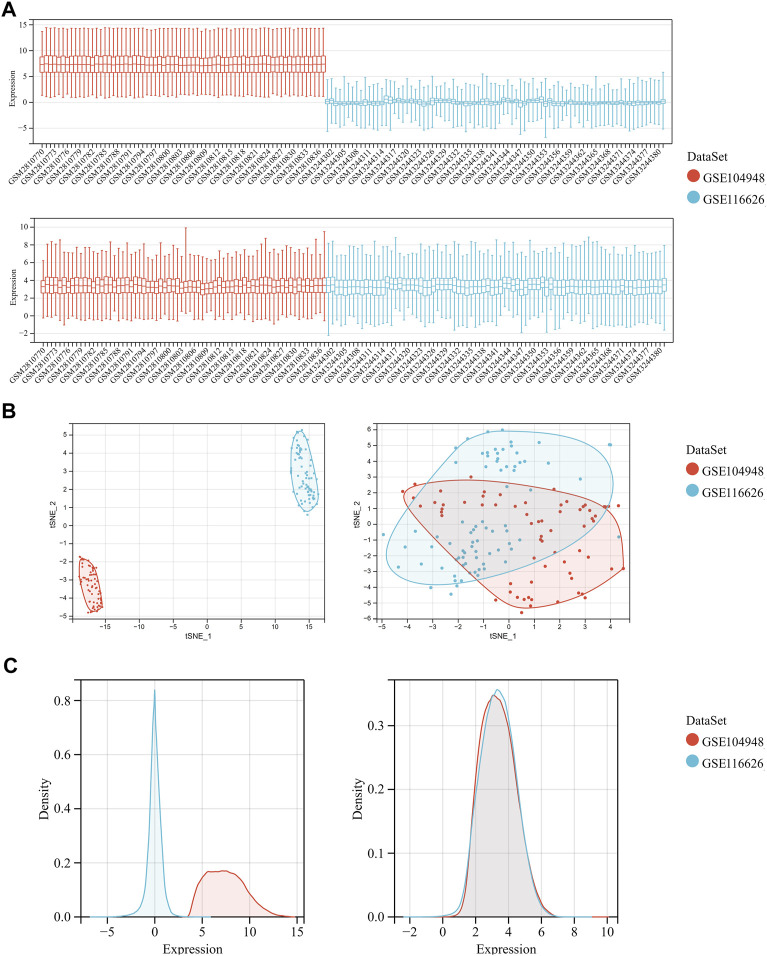
Data preprocessing of GSE104948 and GSE116626. **(A)** Box diagram showing the sample distribution of each data set before batch correction and after batch correction. **(B)** UMAP analysis showing the sample distribution of each data set before batch correction and after batch correction. **(C)** Density map showing the sample distribution of each data set before batch correction and after batch correction.

After merging the GSE104948 and GSE116626 datasets, we removed batch-to-batch variance from the matrix of gene expression (Supplementary File S1, S2). In Figure 2A, the box diagram shows that the sample distribution of each data set is quite different before the batch effect is removed, revealing that the batch effect exists. The sample distributions of the two datasets tend to be consistent after excluding the batch-to-batch variance, and the medians are on the same straight line. Figure 2B depicts UMAP results for multiple datasets with different colors representing different datasets before showing batch deletion. As shown, the two datasets do not intersect with each other and are independent of each other. After removing the batch-to-batch variance, the sample distributions between datasets tend to be consistent. From the density map in Figure 2C, we can observe that there is a great difference in the sample distribution of each data set before excluding the batch effect. The sample distributions between the datasets tend to be consistent after eliminating the batch effect.

### 3.2 Function enrichment analyses of the DEGs

**FIGURE 3:**
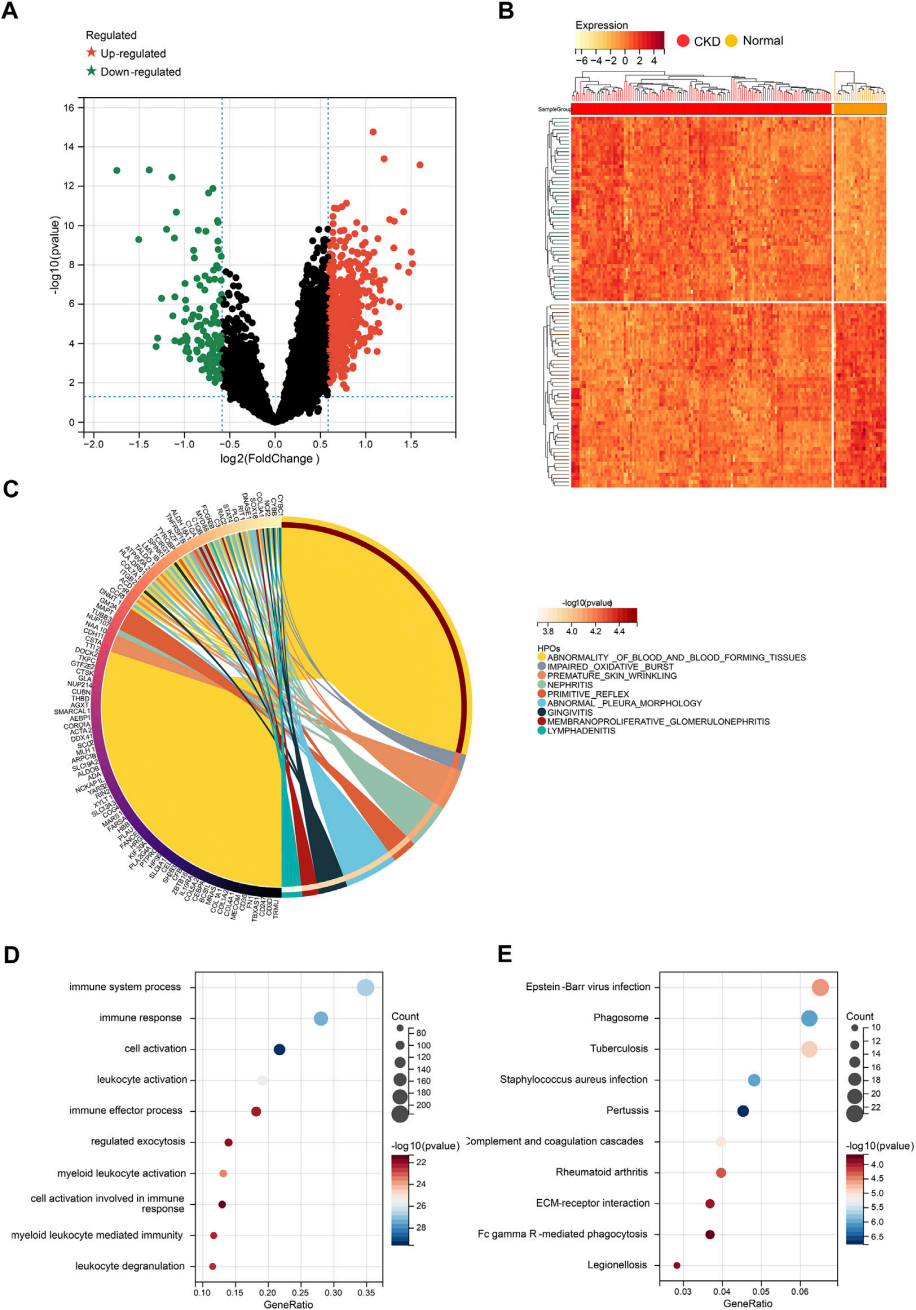
Identification of DEGs for CKD. **(A)** Volcano plots showing DEGs between CKD and normal group. **(B)** Cluster heatmap showing the top 50 significantly upregulated DEGs and the top 50 significantly down-regulated DEGs. **(C)** Top 20 of Human phenotype Ontology analysis. **(D)** Top 20 of GO biological processes analysis. **(E)** Top 20 of KEGG pathway analysis.

After preprocessing the data with R software, we extracted the DEGs in the gene expression matrix. Under the criteria of *p*-value <.05 and | fold-change (FC) | >1.5, 657 genes were identified as DEGs, with 521 genes up-regulated and 136 genes down-regulated (Supplementary File S3). Figures 3A, B show a volcano plot of DEGs and a heatmap of the top 50 DEGs. Next, Human phenotype ontology, GO and KEGG signaling pathway enrichment analyses were performed to dissect the biological functions and signaling pathways involved in 657 selected DEGs (Supplementary Files S4–S6).

The top 10 results of Human phenotype Ontology show that nephritis, membranoproliferative glomerulonephritis, and impaired oxidative burst were significantly enriched (Figure 3C), which indicates the reliability of our data. More importantly, the top 10 GO analysis shows that a large number of biological processes related to immune and inflammatory responses are significantly enriched, including cell activation, immune response, immune system process, leukocyte activation, and myeloid leukocyte activation (Figure 3D). In terms of KEGG Pathway, complement and coagulation cascades, ECM-receptor interaction, and Fc gamma R-mediated phagocytosis are significantly enriched (Figure 3E). The results above strongly suggest that autoimmunity and inflammation play essential roles in the development process of CKD.

### 3.3 Weighted gene co-expression network construction and identification of clinically significant modules

**FIGURE 4:**
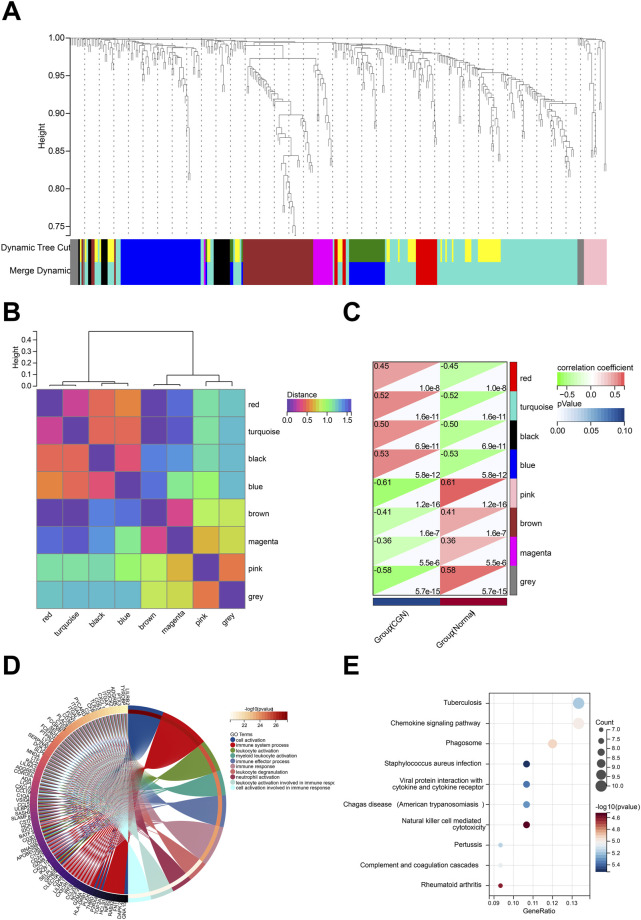
Identification of modules associated with the clinical traits of CKD based on WGCNA analysis. **(A)** Dendrogram of all differentially expressed genes clustered based on a dissimilarity measure (1-TOM). **(B)** Clustering heatmap of module feature vector. **(C)** Heatmap of the correlation between module eigengenes and clinical traits of CKD. **(D)** Top 20 of GO biological processes analysis. **(E)** Top 20 of KEGG pathway analysis.

Based on the screened 657 DEGs expression profile, WGCNA was performed to identify the major modules most associated with CKD (Supplementary Files S7–S10). Eight modules were identified after merging strong association modules with a cluster height limit of 0.25 (Figure 4A). The module feature vector clustering was investigated next, and the results revealed the distance between them (Figure 4B). Furthermore, the correlations between modules and clinical symptoms were explored. The red module (*r* = 0.45, *p* = 1.0e-8), the turquoise module (*r* = 0.52, *p* = 1.6e-11), the black module (*r* = 0.50, *p* = 6.9e-11), and the blue module (*r* = 0.53, *p* = 5.8e-12) are positively correlated with CKD, while the pink module (*r* = −0.61, *p* = 1.2e-16), the brown module (*r* = −0.41, *p* = 1.6e-7), the magenta module (r = −0.36, *p* = 5.5e-6), and the grey module (*r* = −0.58, *p* = 5.7e-15) are negatively correlated with CKD (Figure 4C).

We performed functional enrichment to explore more about the biological functions of the DEGs in eight modules (Supplementary Files S11, S12). The results of GO and KEGG analysis revealed that DEGs in the turquoise module were linked to a large number of biological processes and pathways related to autoimmunity, inflammation, and infection. GO enrichment analysis showed that turquoise module DEG genes have leukocyte activation involved in immune response, cell activation involved in immune response, leukocyte degranulation, and neutrophil activation (Figure 4D). KEGG analysis was associated with Chemokine signaling pathway, Natural killer cell mediated cytotoxicity, Complement and coagulation cascades, and Viral protein interaction with cytokine and cytokine receptor (Figure 4E). According to GS > 0.8 and MM > 0.1, 16 genes in the turquoise module are identified as key genes (MS4A6A, RAC2, GPR65, LYZ, MYO1F, PYCARD, LCP1, CTSS, AOAH, IL10RA, CD53, EVI2A, C1QA, NCF2, PTPRC, MS4A4A).

### 3.4 Hub gene identification

**FIGURE 5:**
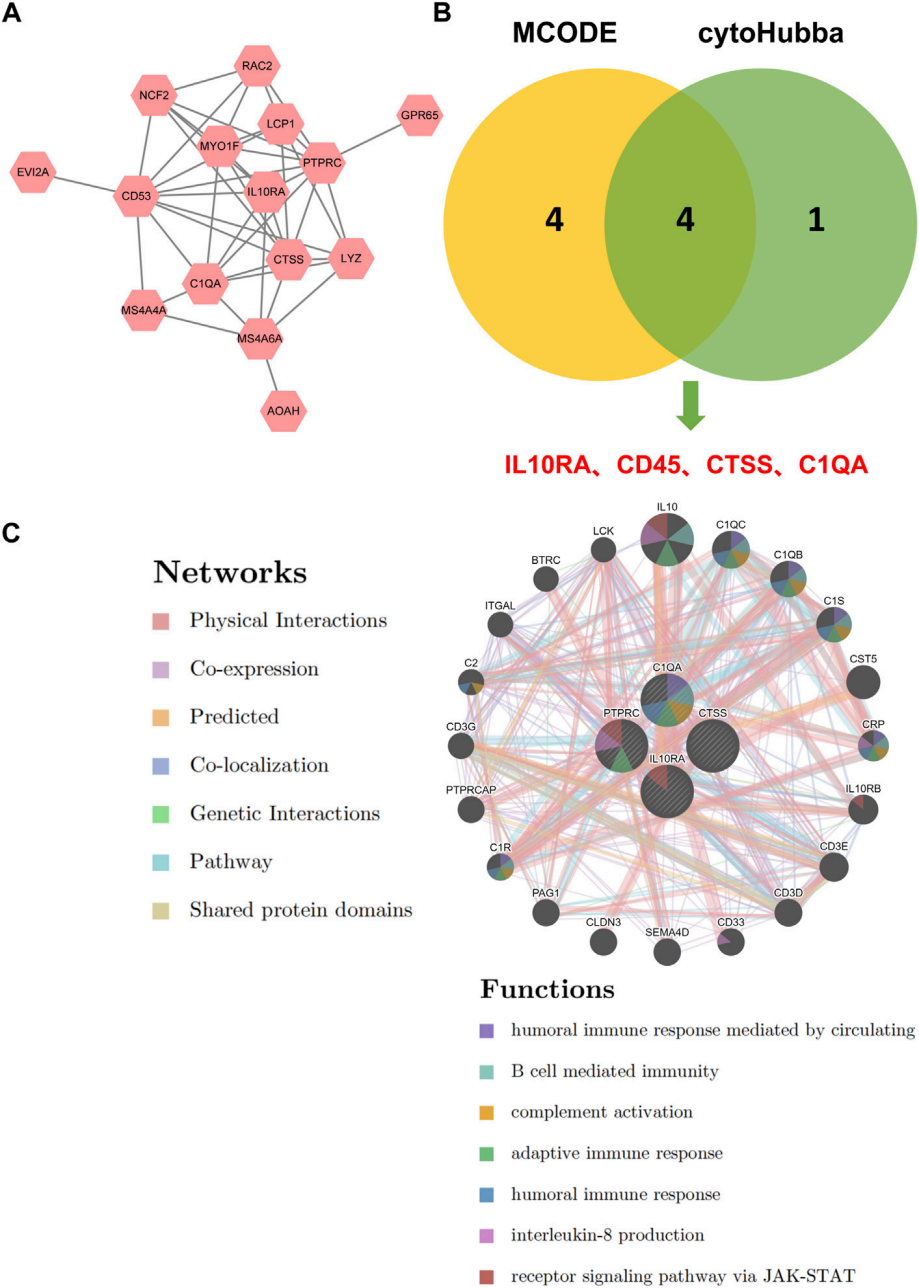
Identification of hub genes for CKD. **(A)** PPI network of key genes. **(B)** The intersection of the key genes calculated by MCC and MCODE is visualized using Venn diagram. **(C)** Hub genes and their co-expression genes were analyzed *via* GeneMANIA.

To further discover CKD-related hub genes and their mechanisms, we mapped the above 16 key genes with high expression in the turquoise module of the CKD group, uploaded them to the online STRING database, and constructed a PPI network (Supplementary File S13). A PPI network with 15 nodes and 43 edges was realized (Figure 5A). Among the 15 nodes, the top 4 genes with a high binding degree were found by Cytoscape (version 3.8.2) MCODE and MCC calculation methods. These genes, which were identified to play hub roles in CKD, are listed as follows: IL10RA, CD45, CTSS, and C1QA (Figure 5B). The specific information of the hub genes is shown in Table 1.

**TABLE 1:** The biological function of biomarkers in detail from GeneCards database.

Gene name	Biological function	Log2FC	*p*-Value
Interleukin-10 receptor alpha subunit (IL10RA)	Participate in IL10-mediated anti-inflammatory functions	1.515	8.73E-09
	Limit excessive tissue disruption caused by inflammation		
Leukocyte common antigen/protein tyrosine phosphatase receptor type C(CD45)	Regulate cell growth, differentiation, mitosis, and oncogenic transformation	0.768	6.54E-06
	Regulate of T- and B-cell antigen receptor signaling		
Cysteine protease cathepsin S(CTSS)	Remodel components of the extracellular matrix	0.951	1.20E-08
	Participate in the pathology of many inflammatory and autoimmune diseases		
Complement component 1, Q subcomponent, alpha polypeptide (C1QA)	Be associated with lupus erythematosus and glomerulonephritis	1.074	4.12E-07
	Lead to immunodeficiency due to complement deficiency		

Next, we explored the co-expression networks and potential functions of hub genes according to the GeneMANIA database (Figure 5C) (Supplementary File S14). They revealed the sophisticated PPI networks with the protein domains of 0.60%, pathway of 1.88%, genetic interactions of 2.87%, co-localization of 3.64%, predicted of 5.37%, co-expression of 8.01%, physical interactions of 77.64%. Function analysis indicated that they are mainly related to a variety of immune and inflammatory pathways, including humoral immune response mediated by circulation, B cell mediated immunity, complement activation, adaptive immune response, humoral immune response, interleukin-8 production, and receptor signaling pathway *via* JAK-STAT, revealing their essential role in contributing to CKD pathogenesis.

### 3.5 Diagnostic value and validation of hub gene on CKD

**FIGURE 6:**
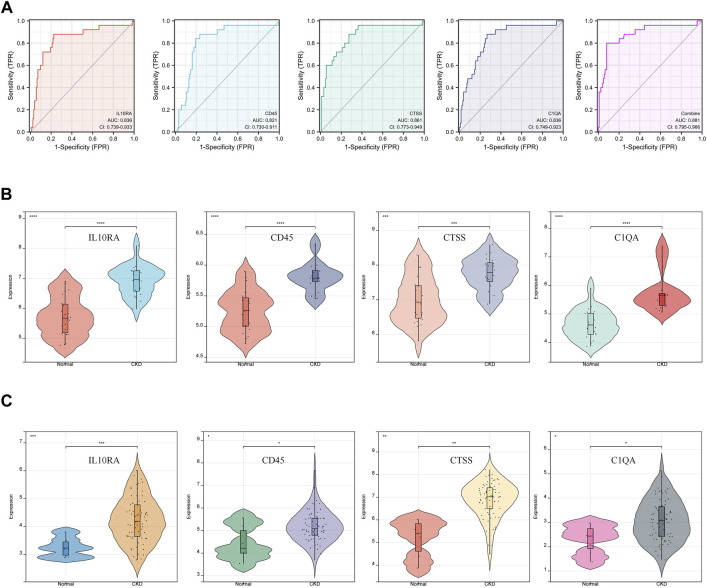
Diagnostic effectiveness and dataset validation of the hub genes for CKD. **(A)** ROC curves to assess the diagnostic efficacy of hub genes. **(B)** Data validation of hub genes by GSE93798. **(C)** Data validation of hub genes by GSE104066.

We conducted ROC analysis to study the relationship between hub gene expression and the prognosis of CKD patients (Supplementary File S15). An AUC greater than 0.800 is considered to have excellent specificity and sensitivity for the diagnosis of CKD. As shown in Figure 6A, the AUC value of IL10RA was 0.821 (95% CI: 0.730-0.911), CD45 was 0.836 (95% CI: 0.739-0.933), CTSS was 0.861 (95% CI: 0.773-0.949), and C1QA was 0.836 (95% CI: 0.749-0.923). More importantly, the combination of all 4 hub genes is 0.881 (95% CI: 0.795-0.966). The results showed that IL10RA, CD45, CTSS, and C1QA have high diagnostic value.

Furthermore, two new CKD-related datasets, including GSE93798 (Figure 6B) and GSE104066 (Figure 6C), validated the above four hub DEGs (Supplementary File S16). Through verification, the mRNA expression of each hub gene was significantly overexpressed in CKD, compared with the control. The validation results fully support the assumption that IL10RA, CD45, CTSS, and C1QA may be diagnostic markers of CKD.

### 3.6 Increased expression of hub gene in kidney tissues of CKD

**FIGURE 7:**
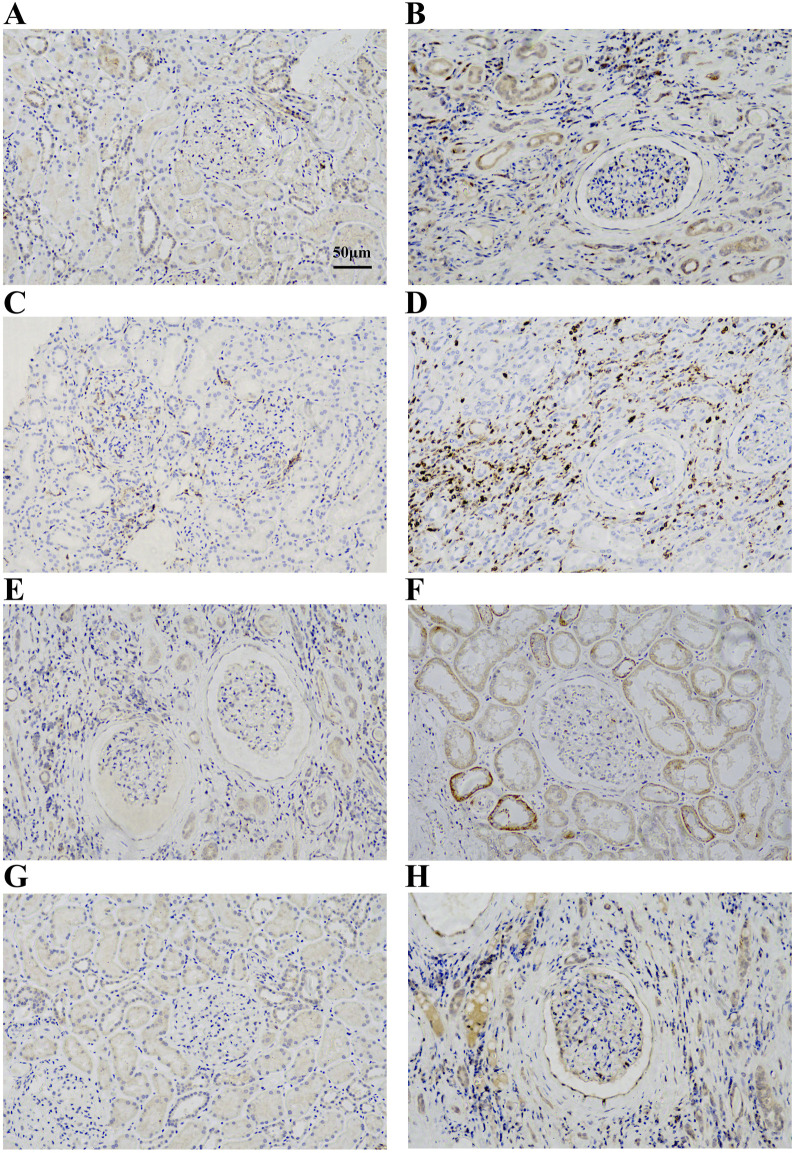
**(A)** Immunohistochemical analysis of IL10RA expression in control group. **(B)** Immunohistochemical analysis of IL10RA expression in CKD group. **(C)** Immunohistochemical analysis of CD45 expression in control group. **(D)** Immunohistochemical analysis of CD45 expression in CKD group. **(E)** Immunohistochemical analysis of CTSS expression in control group. **(F)** Immunohistochemical analysis of CTSS expression in CKD group. **(G)** Immunohistochemical analysis of C1QA expression in control group. **(H)** Immunohistochemical analysis of C1QA expression in CKD group.

To verify the expression of IL10RA, CD45, CTSS, and C1QA in CKD, we treated kidney tissues with IHC and found that IL10RA, CD45, CTSS, and C1QA were all highly expressed in the CKD tissues compared with the control subjects, which is consistent with our bioinformatics prediction (Figures 7A–H).

### 3.7 Immune cell infiltration analysis

**FIGURE 8:**
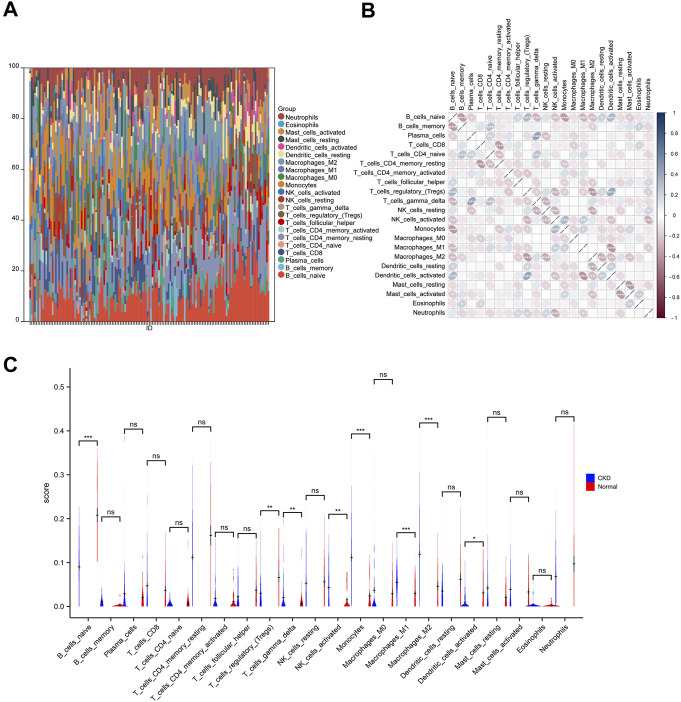
Immune infiltration analysis of CKD. **(A)** The ratio of 22 immune cells of each sample of CKD. **(B)** The correlation between each of immune cells. **(C)** The proportion of immune cells in CKD and control.

To examine differences in immune patterns between CKD and normal tissues, the matrix of gene expression estimated the infiltration ratio of 22 immune cells using the CIBERSORT method (Supplementary Files S17–S19). In each sample, a histogram depicted the composition of 22 types of immune cells (Figure 8A). Colors on every histogram exhibit the percentages of immune cells, with a sum of 1 for each sample. The results indicated that naive B cells (137), neutrophils (136), M1 macrophages (133), M2 macrophages (131), and resting CD4 memory T cells (129) were the most abundant immuno-infiltrating cells in all 149 samples. In the following study, 22 kinds of immune cells in CKD samples were evaluated for their correlation (Figure 8B). The correlation heat map of 22 immune cells showed that naive B cells (GBD 2016 Causes of Death Collaborators, 2017), regulatory T cells (Dekker et al., 2017), monocytes (Zimmermann et al., 1999), M2 macrophages (Zoccali et al., 2006), and activated NK cells (Honda et al., 2006) are associated with most immune cells. However, activated CD4 memory T cells (Holle et al., 2022), M0 macrophages (Holle et al., 2022), CD8 T cells (Quon et al., 2011), resting CD4 memory T cells (Quon et al., 2011), and eosinophils (Quon et al., 2011) are only associated with a few immune cells. Violin plots of the difference in immune cell infiltration indicated that, compared with the normal control sample, gamma delta T cells, activated NK cells, monocytes, M0 macrophages, and M1 macrophages infiltrated more, while naive B cells, regulatory T cells and activated dendritic cells infiltrated less (Figure 8C).

### 3.8 Correlation between hub genes and immune cells

**FIGURE 9:**
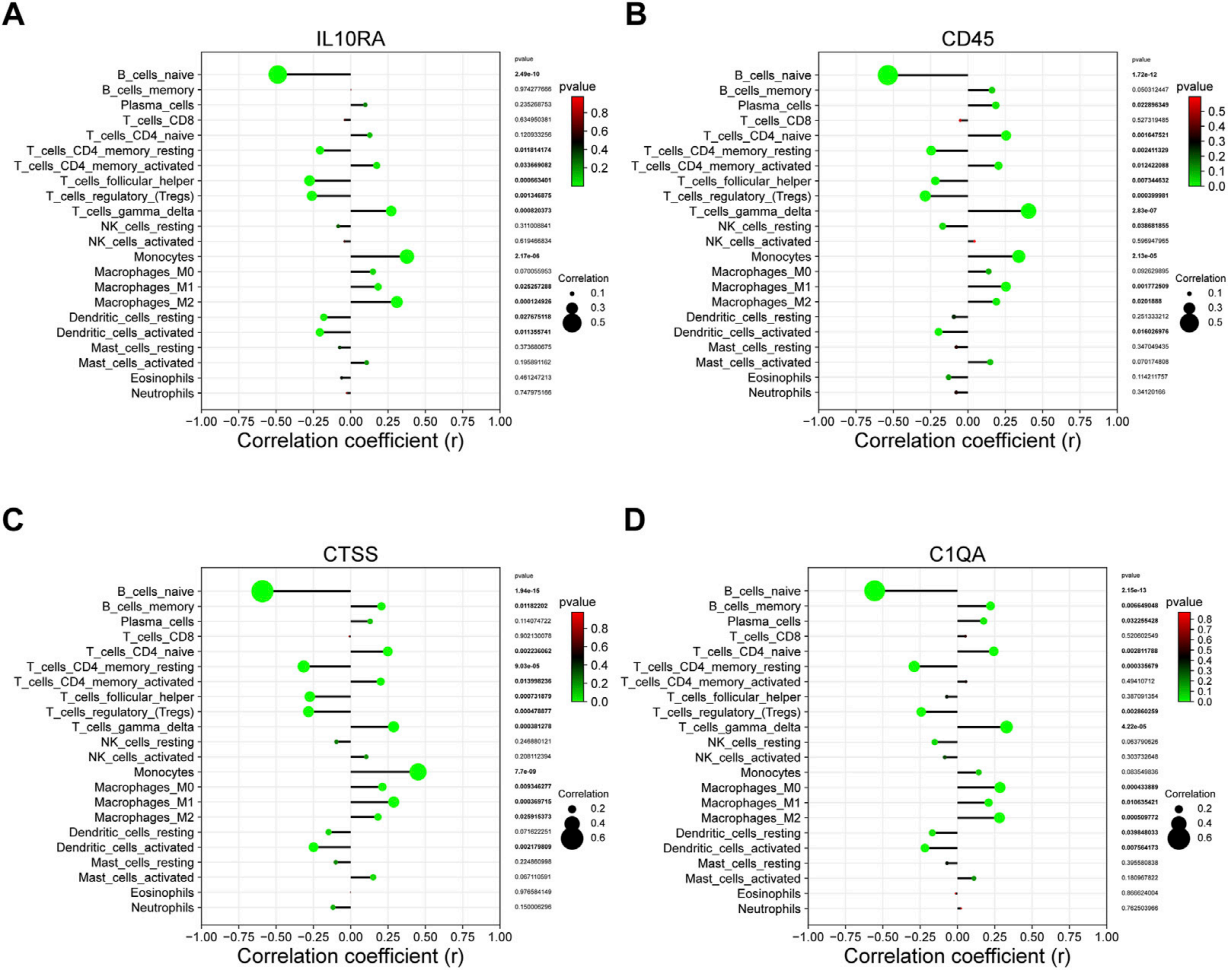
The correlation between the hub gene and the immune cell. **(A)** IL10RA; **(B)** CD45; **(C)** CTSS; **(D)** C1QA.

We further explored whether there is a potential correlation between immune cell abundance and hub gene expression using Pearson’s correlation analysis (Supplementary File S20). As shown in Figures 9A–D, a total of seven immune cell populations that are related to all four core genes, of which naive B cells, resting memory CD4 T cells, regulatory T cells, and activated dendritic cells were statistically negatively with IL10RA, CD45, CTSS, and C1QA, while gamma delta T cells, monocytes, M0 macrophages, and M1 macrophages were positively correlated with them, suggesting they may play essential roles in CKD development.

### 3.9 GSEA of IL10RA

**FIGURE 10:**
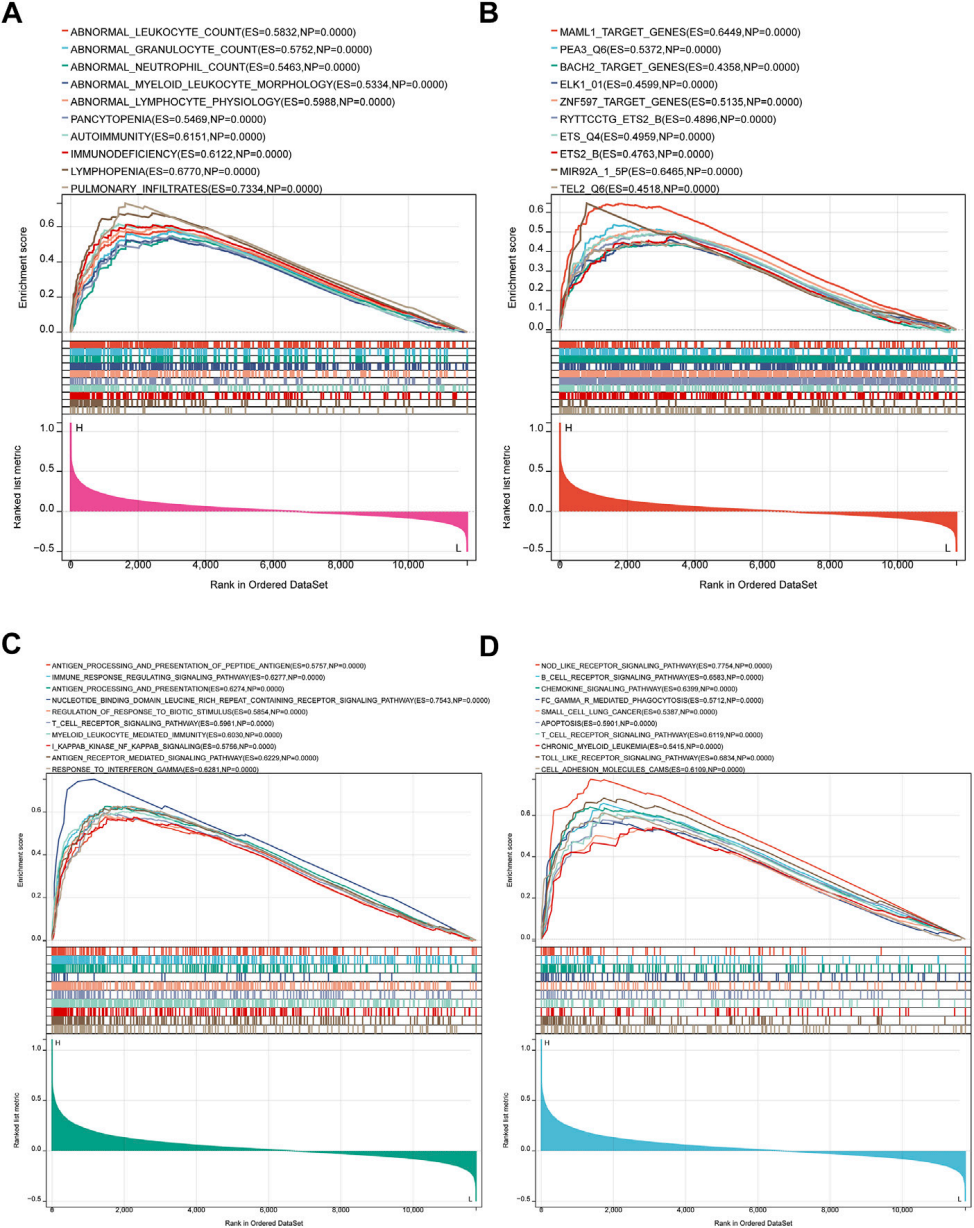
Result of Gene Set Enrichment Analysis of IL10RA. **(A)** Regulatory target genes enriched by IL10RA. **(B)** Biological processes enriched by IL10RA. **(C)** KEGG pathways by IL10RA. **(D)** Human phenotype Ontology enriched by IL10RA.

Since IL10RA plays an important role in immune infiltration, and the log2FC of IL10RA is the largest of the central genes, we performed an IL10RA analysis using the GSEA method to gain insight into the biological processes and predict the potential signal pathways of IL10RA expression in CKD (Supplementary Files S21–S24). The top 10 results of MSigDB C5 Human phenotype Ontology showed IL10RA was involved in abnormalities in various immune cells, including abnormal leukocyte, abnormal granulocyte, abnormal neutrophil, abnormal myeloid leukocyte morphology, and abnormal lymphocyte physiology (Figure 10A). In addition, high levels of IL10RA may affect several manipulated downstream potential genes, including MAML1, PEA3, BACH2, ELK1, ZNF597, ETS2, MIR92A, and TEL2 (Figure 10B).

More importantly, the top 10 results of MSigDB C5 GO biological processes showed IL10RA regulated a large number of immune system responses, including antigenic processing and presentation of polypeptide antigen, immune response regulating signal pathway, regulation of response to biotic stimulus, T cell receptor signaling pathway, myeloid leukocyte mediated immunity, I kappaB kinase NF kappaB signaling, and response to interferon gamma (Figure 10C). Meanwhile, MSigDB C2 KEGG gene sets found that in addition to B cell receptor signaling pathway, T cell receptor signaling pathway, and Fc gamma R-mediated phagocytosis, IL10RA was also related to a large number of inflammation-related pathways, including chemokine signaling pathway, apoptosis, Nod like receptor signaling pathway, Toll like receptor signaling pathway, and cell adhesion molecules cams. Thus, the above results suggest that the immune and inflammatory responses play essential roles in IL10RA contributing to the CKD pathogenesis (Figure 10D).

### 3.10 Drug-Gene interaction and molecular docking analyses of IL10RA

**FIGURE 11:**
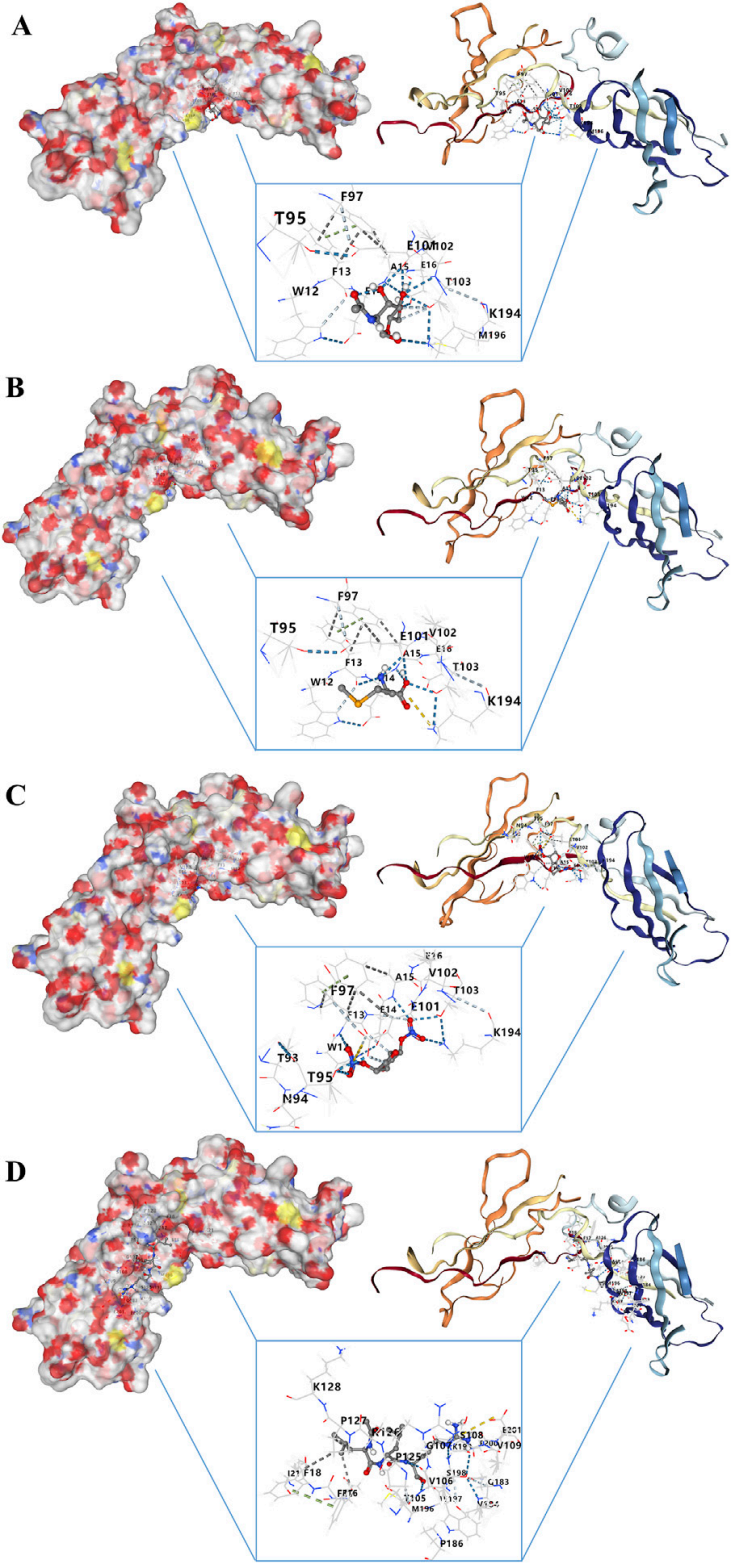
Molecular docking analysis of drug–gene interaction. **(A)** Molecular docking between IL10RA and chitin. **(B)** Molecular docking between IL10RA and selenomethioni. **(C)** Molecular docking between IL10RA and isosorbide din. **(D)** Molecular docking between IL10RA and leupeptin.

Searching for targeted drugs for IL10RA provides a new strategy for potential drug therapy for CGN. Based on the STITCH database, we obtained 4 small molecular drugs, including chitin, selenomethioni, leupeptin, and isosorbide din (Supplementary File S25). Then, the above 4 bioactive compound ligands were docked with the protein IL10RA to evaluate the binding potential. As shown in Figures 11A–D, the docking 3D model of protein IL10RA and four small molecules drugs with the firmest binding, showing their potential to alleviate or even reverse CKD development (Supplementary File S26).

## 4 Discussion

The kidneys are highly susceptible to excessive inflammatory responses due to the system’s autoimmunity (Kurts et al., 2013). In particular, renal tubular epithelial cells (TECs), which play critical roles as antigen-presenting cells, interact directly with neutrophils, monocytes, and T lymphocytes through the activation of cell adhesion molecules that are caused by tubular injuries. In addition, damage usually spreads to distant organs (including the heart, liver, lungs) after kidney injury, which is a vicious circle (Kosugi and Sato, 2012; Cantaluppi et al., 2014). It has been suggested that cytokines produced by circulating immune cells and damaged organs may mediate kidney-to-kidney crosstalk (Lv et al., 2020). Persistent renal injury can lead to irreversible pathological changes, such as glomerular aging, interstitial fibrosis, etc., regardless of the primary disease processes, and finally lead to the development of CKD (Livingston et al., 2016). Therefore, the prevention of renal immunity and inflammation is crucial to decrease mortality and morbidity after renal injury. The ideal approach to identifying appropriate treatments for this type of disease includes early diagnosis and treatment of CKD, as well as identification of inflammation induced by diverse potential mechanisms and immune system involvement. As a result, identifying the potential biomarkers associated with CKD development is an effective method for preventing and treating CKD.

In this study, we screened 1178 differentially expressed genes (DEGs) and found 657 genes were upregulated and 521 were downregulated. Subsequent GO enrichment analysis showed a large number of biological processes related to immune and inflammatory responses (immune response, immune system process, myeloid leukocyte activation) are significantly enriched, while KEGG enrichment analysis showed some correlation with complement and coagulation cascades and ECM-receptor interaction, along with Fc gamma R-mediated phagocytosis. Besides, Human phenotype Ontology further confirms the results above. The DEGs were mainly mapped in nephritis, membranoproliferative glomerulonephritis, and impaired oxidative burst. This suggests that the DEGs could have a function in participating in the pathogenesis of CKD.

Next, we identified eight CKD-related modules based on WGCNA analysis. DEGs in the turquoise module were found to be involved in plenty of inflammation and immune-related biological processes and pathways. Furthermore, 16 key genes in the turquoise module were screened according to MM > 0.8 and GS > 0.1. Finally, we obtained four hub genes through PPI network and interaction analysis, namely, IL10RA, CD45, CTSS, and C1QA (all upregulated genes). Many of them have been implicated in immune and inflammatory responses in other diseases, but fewer have been mentioned in the development of CKD. IL10RA (interleukin-10 receptor alpha subunit) is a protein-coding gene that mediates interleukin-10 immunosuppressive signaling. Mutations in the gene that encodes the subunit protein of IL10R are associated with a hyper-inflammatory immune response in the gut (Glocker et al., 2009; Shouval et al., 2014; Oh et al., 2019; Liu et al., 2021). CD45/PTPRC (leukocyte common antigen/protein tyrosine phosphatase receptor type C) is a transmembrane glycoprotein expressed on almost all hematopoietic cells except mature red blood cells, and is an essential regulator of T and B cell antigen receptor-mediated activation (Al Barashdi et al., 2021). CTSS (cysteine protease cathepsin S) regulates biological activities in and out of cells, including immunity and inflammation (Toyama et al., 2020). There is evidence that CTSS may be beneficial in treating renal fibrosis. Among its functions, CTSS may regulate fibrosis *via* the TGF/SMAD pathway and influence ECM deposition as well as epithelial-mesenchymal transition (EMT) (Yao et al., 2019). The C1QA (complement component 1, Q subcomponent, alpha polypeptide) encodes C1q, a major component of serum complement, which identifies immune complexes and initiates the classical complement pathway (Lao et al., 2008). C1QA deficiency is associated with lupus erythematosus and glomerulonephritis (Held et al., 2008; Namjou et al., 2009). Furthermore, the ROC curve analysis and two CKD validated datasets verified the reliability of their diagnostic value. More importantly, a significant increase in IL10RA, CD45, CTSS, and C1QA was observed by IHC in clinical CKD patients.

In order to fully comprehend the dysfunctional inflammatory cells in CKD, an immune infiltration analysis was performed. It was found that CKD tissue owned a higher gamma delta T cells, activated NK cells, monocytes, M1 macrophages, and M2 macrophages, but relatively lower ones of naive B cells, regulatory T cells and activated dendritic cells. Additionally, our study revealed that major infiltration cells were statistically related to each hub gene (IL10RA, CD45, CTSS, and C1QA). In particular, naive B cells, resting memory CD4 T cells, regulatory T cells, and activated dendritic cells were statistically negatively correlated with all hub genes, and gamma delta T cells, monocytes, M1 macrophages, and M2 macrophages were positively correlated with them. Accordingly, they may be associated with the dysfunction of inflammatory cells in CKD and may have a pivotal role in its immunomodulation. Tregs (regulatory T cells) are a type of CD4^+^ T cells that suppress the immune response of effector T cells, B cells, and innate immune cells. Renal and systemic inflammatory immunity are restricted by multiple mechanisms of Tregs (Ghali et al., 2016). Recent studies suggest that Tregs numbers are decreased and their regulatory functions may be impaired in kidney disease (Hu et al., 2016). Recent research has shown that renal macrophages are heterogeneous with multiple functions, including remove adherent pathogen, maintain immune tolerance, initiate and regulate inflammatory response, promote renal fibrosis, and degrade the ECM (Wen et al., 2021). The majority of tissue macrophages are derived from monocytes. The bone marrow produces the cells of the monocytes/macrophages system that reach organs through the blood, migrate through the microvessels through the venules, and further differentiate into macrophages, specific organ tissues. Activated monocytes/macrophages enhance autoimmune responses in mice and other species (Steiniger et al., 2001). The macrophage can be divided into two distinct phenotypes: classical macrophage activation (M1 macrophage), which releases inflammatory cytokines and fibrosis; and activated macrophage (M2 macrophage), which is associated with immune regulation and tissue remodeling function (Liu et al., 2014). The function of dendritic cells in regulating T-cell activation and tolerance is the focus of most research on these cells as professional antigen-presenting cells (Lu, 2012). Research has shown that dendritic cells are crucial in initiating innate immunity and orchestrating inflammation following kidney ischemia-reperfusion (Li and Okusa, 2010). They are responsible for inducing and regulating inflammatory responses in response to fluid that is freely filtered and protecting the kidney from infection (Rogers et al., 2014). In spite of this, there are few studies that explore the relationship between CKD and naive B cells, resting memory CD4 T cells, and gamma delta T cells, which might be an interesting finding.

We chose IL10RA, which obtained the highest expression level in hub genes, to do further analysis. GSEA analysis showed IL10RA was involved in abnormalities in various immune cells and regulated a major number of immune system responses and inflammatory pathways, such as NF-kappaB signal pathways, Nod like receptor signal pathways, Toll like receptor signal pathways, and apoptosis, demonstrating that IL10RA may be a potential biomarker for CKD diagnosis and prognosis. NF-κB signal pathways have long been recognized as typical pro-inflammatory pathways and these pathways are activated by inflammatory cytokines, such as TNF-α and IL-1β (Lawrence, 2009). Activation of the NF-κB signaling pathway has been implicated in the pathogenesis of a variety of human diseases, including brain and kidney diseases, and plays an important role in the initiation and progression of inflammation (White et al., 2020). The NOD-like receptor (NLR) family of proteins is a group of pattern recognition receptors (PRRs) known to mediate the initial innate immune response to cellular injury and stress, whose activation not only occurs in immune cells, but also in residential cells such as endothelial cells and podocytes in the glomeruli (Conley et al., 2017; Platnich and Muruve, 2019). Studies have shown that activation of the NLRP3 inflammasome may lead to glomerular injury and the development of ESRD, thereby triggering inflammation and other cellular damage (Komada and Muruve, 2019). Similarly, the toll-like receptor family (TLRs) serves a key manipulative role in the innate immune system, and recent research shows the transduction of TLR signaling is related to the inflammatory response to various exogenous and endogenous stimuli in the kidney (Garibotto et al., 2017). In addition to their established roles in host defense, TLRs also play new roles, controlling body balance, disrupting, and repairing wounds (Ramnath et al., 2017). As an activated form of programmed cell death, apoptosis keeps the body environment stable (Zhao et al., 2019). Genes directly control cell apoptosis and proliferation, which ensure dynamic equilibrium of the body’s cells (Guan et al., 2019). Apoptosis has been found to be an essential component of glomerular remodeling, mediating the excessive regression of glomerular cells during CGN repair (Shimizu et al., 1996; Hughes and Savill, 2005). Moreover, we further identified four potential therapeutic drugs targeting IL10RA, which provides a possible therapeutic strategy for CKD. Molecular docking revealed that the exact molecular binding makes this relationship more reliable.

## 5 Conclusion

In sum, we identified 4 hub genes, IL10RA, CD45, CTSS, and C1QA, from CKD-related genes, which are mainly involved in the inflammatory response and maladjustment of immune cells in CKD. In particular, IL10RA might play a role in abnormalities in various immune cells and the activation of inflammation-related pathways. Therefore, IL10RA and its related hub molecules might be potential key biomarkers in the development of CKD, and our study would provide a new perspective on the etiopathogenesis and therapeutic programs of CKD.

## Data Availability

The datasets presented in this study can be found in online repositories. The names of the repository/repositories and accession number(s) can be found in the article/Supplementary Material.

## References

[B1] Al Barashdi M. A. Ali A. McMullin M. F. Mills K. (2021). Protein tyrosine phosphatase receptor type C (PTPRC or CD45). J. Clin. Pathol. 74 (9), 548–552. 10.1136/jclinpath-2020-206927 34039664 PMC8380896

[B2] Aveles P. R. Criminácio C. R. Gonçalves S. Bignelli A. T. Claro L. M. Siqueira S. S. (2010). Association between biomarkers of carbonyl stress with increased systemic inflammatory response in different stages of chronic kidney disease and after renal transplantation. Nephron Clin. Pract. 116 (4), c294–c299. 10.1159/000318792 20639676

[B3] Brix S. R. Noriega M. Herden E. M. Goldmann B. Langbehn U. Busch M. (2018). Organisation of lymphocytic infiltrates in ANCA-associated glomerulonephritis. Histopathology 72 (7), 1093–1101. 10.1111/his.13487 29453894

[B4] Cantaluppi V. Quercia A. D. Dellepiane S. Ferrario S. Camussi G. Biancone L. (2014). Interaction between systemic inflammation and renal tubular epithelial cells. Nephrol. Dial. Transpl. 29 (11), 2004–2011. 10.1093/ndt/gfu046 24589723

[B5] Chen B. Khodadoust M. S. Liu C. L. Newman A. M. Alizadeh A. A. (2018). Profiling tumor infiltrating immune cells with CIBERSORT. Methods Mol. Biol. 1711, 243–259. 10.1007/978-1-4939-7493-1_12 29344893 PMC5895181

[B6] Cippà P. E. Liu J. Sun B. Kumar S. Naesens M. McMahon A. P. (2019). A late B lymphocyte action in dysfunctional tissue repair following kidney injury and transplantation. Nat. Commun. 10 (1), 1157. 10.1038/s41467-019-09092-2 30858375 PMC6411919

[B7] Cohen C. D. Calvaresi N. Armelloni S. Schmid H. Henger A. Ott U. (2005). CD20-positive infiltrates in human membranous glomerulonephritis. J. Nephrol. 18 (3), 328–333. 16013025

[B8] Conley S. M. Abais J. M. Boini K. M. Li P. L. (2017). Inflammasome activation in chronic glomerular diseases. Curr. Drug Targets 18 (9), 1019–1029. 10.2174/1389450117666160817103435 27538510 PMC5893309

[B9] Dekker M. J. Marcelli D. Canaud B. J. Carioni P. Wang Y. Grassmann A. (2017). Impact of fluid status and inflammation and their interaction on survival: A study in an international hemodialysis patient cohort. Kidney Int. 91 (5), 1214–1223. 10.1016/j.kint.2016.12.008 28209335

[B10] Dubey L. K. Karempudi P. Luther S. A. Ludewig B. Harris N. L. (2017). Interactions between fibroblastic reticular cells and B cells promote mesenteric lymph node lymphangiogenesis. Nat. Commun. 8 (1), 367. 10.1038/s41467-017-00504-9 28848229 PMC5573728

[B11] Franceschi C. Capri M. Monti D. Giunta S. Olivieri F. Sevini F. (2007). Inflammaging and anti-inflammaging: A systemic perspective on aging and longevity emerged from studies in humans. Mech. Ageing Dev. 128 (1), 92–105. 10.1016/j.mad.2006.11.016 17116321

[B12] Garibotto G. Carta A. Picciotto D. Viazzi F. Verzola D. (2017). Toll-like receptor-4 signaling mediates inflammation and tissue injury in diabetic nephropathy. J. Nephrol. 30 (6), 719–727. 10.1007/s40620-017-0432-8 28933050

[B13] Gbd 2015 Mortality and Causes of Death Collaborators. Global, regional, and national life expectancy, all-cause mortality, and cause-specific mortality for 249 causes of death, 1980-2015: A systematic analysis for the global burden of disease study 2015. Lancet. 2016;388(10053):1459–1544. 10.1016/S0140-6736(16)31012-1 27733281 PMC5388903

[B14] Gbd 2016 Causes of Death Collaborators. Global, regional, and national age-sex specific mortality for 264 causes of death, 1980-2016: A systematic analysis for the global burden of disease study 2016. Lancet. 2017;390(10100):1151–1210. 10.1016/S0140-6736(17)32152-9 28919116 PMC5605883

[B15] Ghali J. R. Wang Y. M. Holdsworth S. R. Kitching A. R. (2016). Regulatory T cells in immune-mediated renal disease. Nephrol. Carlt. 21 (2), 86–96. 10.1111/nep.12574 26206106

[B16] Glassock R. Delanaye P. El Nahas M. (2015). An age-calibrated classification of chronic kidney disease. JAMA 314 (6), 559–560. 10.1001/jama.2015.6731 26023760

[B17] Glocker E. O. Kotlarz D. Boztug K. Gertz E. M. Schaffer A. A. Noyan F. (2009). Inflammatory bowel disease and mutations affecting the interleukin-10 receptor. N. Engl. J. Med. 361 (21), 2033–2045. 10.1056/NEJMoa0907206 19890111 PMC2787406

[B18] Glorieux G. Helling R. Henle T. Brunet P. Deppisch R. Lameire N. (2004). *In vitro* evidence for immune activating effect of specific AGE structures retained in uremia. Kidney Int. 66 (5), 1873–1880. 10.1111/j.1523-1755.2004.00961.x 15496158

[B19] Glorieux G. L. Dhondt A. W. Jacobs P. Van Langeraert J. Lameire N. H. De Deyn P. P. (2004). *In vitro* study of the potential role of guanidines in leukocyte functions related to atherogenesis and infection. Kidney Int. 65 (6), 2184–2192. 10.1111/j.1523-1755.2004.00631.x 15149331

[B20] Guan X. Lu J. Sun F. Li Q. Pang Y. (2019). The molecular evolution and functional divergence of lamprey programmed cell death genes. Front. Immunol. 10, 1382. 10.3389/fimmu.2019.01382 31281315 PMC6596451

[B21] Harari-Steinberg O. Metsuyanim S. Omer D. Gnatek Y. Gershon R. Pri-Chen S. (2013). Identification of human nephron progenitors capable of generation of kidney structures and functional repair of chronic renal disease. EMBO Mol. Med. 5 (10), 1556–1568. 10.1002/emmm.201201584 23996934 PMC3799579

[B22] Held K. Thiel S. Loos M. Petry F. (2008). Increased susceptibility of complement factor B/C2 double knockout mice and mannan-binding lectin knockout mice to systemic infection with Candida albicans. Mol. Immunol. 45 (15), 3934–3941. 10.1016/j.molimm.2008.06.021 18672286

[B23] Holle J. Bartolomaeus H. Löber U. Behrens F. Bartolomaeus T. U. P. Anandakumar H. (2022). Inflammation in children with CKD linked to gut dysbiosis and metabolite imbalance. J. Am. Soc. Nephrol. 33, 2259–2275. ASN.2022030378. 10.1681/ASN.2022030378 35985814 PMC9731629

[B24] Honda H. Qureshi A. R. Heimbürger O. Barany P. Wang K. Pecoits-Filho R. (2006). Serum albumin, C-reactive protein, interleukin 6, and fetuin a as predictors of malnutrition, cardiovascular disease, and mortality in patients with ESRD. Am. J. Kidney Dis. 47 (1), 139–148. 10.1053/j.ajkd.2005.09.014 16377395

[B25] Hu M. Wang Y. M. Wang Y. Zhang G. Y. Zheng G. Yi S. (2016). Regulatory T cells in kidney disease and transplantation. Kidney Int. 90 (3), 502–514. 10.1016/j.kint.2016.03.022 27263492

[B26] Hughes J. Savill J. S. (2005). Apoptosis in glomerulonephritis. Curr. Opin. Nephrol. Hypertens. 14 (4), 389–395. 10.1097/01.mnh.0000172728.82993.4e 15931010

[B27] Jankowski J. Floege J. Fliser D. Böhm M. Marx N. (2021). Cardiovascular disease in chronic kidney disease: Pathophysiological insights and therapeutic options. Circulation 143 (11), 1157–1172. 10.1161/CIRCULATIONAHA.120.050686 33720773 PMC7969169

[B28] Johnson W. E. Li C. Rabinovic A. (2007). Adjusting batch effects in microarray expression data using empirical Bayes methods. Biostatistics 8 (1), 118–127. 10.1093/biostatistics/kxj037 16632515

[B29] Karuppasamy M. P. Venkateswaran S. Subbiah P. (2020). PDB-2-PBv3.0: An updated protein block database. J. Bioinform Comput. Biol. 18 (2), 2050009. 10.1142/S0219720020500092 32404014

[B30] Kim S. Chen J. Cheng T. Gindulyte A. He J. He S. (2021). PubChem in 2021: New data content and improved web interfaces. Nucleic Acids Res. 49 (D1), D1388–D1395. 10.1093/nar/gkaa971 33151290 PMC7778930

[B31] Komada T. Muruve D. A. (2019). The role of inflammasomes in kidney disease. Nat. Rev. Nephrol. 15 (8), 501–520. 10.1038/s41581-019-0158-z 31164720

[B32] Kosugi T. Sato W. (2012). Midkine and the kidney: Health and diseases. Nephrol. Dial. Transpl. 27 (1), 16–21. 10.1093/ndt/gfr652 22167595

[B33] Kratz A. Campos-Neto A. Hanson M. S. Ruddle N. H. (1996). Chronic inflammation caused by lymphotoxin is lymphoid neogenesis. J. Exp. Med. 183 (4), 1461–1472. 10.1084/jem.183.4.1461 8666904 PMC2192477

[B34] Krautler N. J. Kana V. Kranich J. Tian Y. Perera D. Lemm D. (2012). Follicular dendritic cells emerge from ubiquitous perivascular precursors. Cell 150 (1), 194–206. 10.1016/j.cell.2012.05.032 22770220 PMC3704230

[B35] Kreimann K. Jang M. S. Rong S. Greite R. von Vietinghoff S. Schmitt R. (2020). Ischemia reperfusion injury triggers CXCL13 release and B-cell recruitment after allogenic kidney transplantation. Front. Immunol. 11, 1204. 10.3389/fimmu.2020.01204 32849490 PMC7424013

[B36] Kurts C. Panzer U. Anders H. J. Rees A. J. (2013). The immune system and kidney disease: Basic concepts and clinical implications. Nat. Rev. Immunol. 13 (10), 738–753. 10.1038/nri3523 24037418

[B37] Lam W. W. Siu S. W. (2017). PyMOL mControl: Manipulating molecular visualization with mobile devices. Biochem. Mol. Biol. Educ. 45 (1), 76–83. 10.1002/bmb.20987 27292587

[B38] Lao H. H. Sun Y. N. Yin Z. X. Wang J. Chen C. Weng S. P. (2008). Molecular cloning of two C1q-like cDNAs in Mandarin fish *Siniperca chuatsi* . Vet. Immunol. Immunopathol. 125 (1-2), 37–46. 10.1016/j.vetimm.2008.05.004 18571244

[B39] Lawrence T. (2009). The nuclear factor NF-kappaB pathway in inflammation. Cold Spring Harb. Perspect. Biol. 1 (6), a001651. 10.1101/cshperspect.a001651 20457564 PMC2882124

[B40] Lee Y. Chin R. K. Christiansen P. Sun Y. Tumanov A. V. Wang J. (2006). Recruitment and activation of naive T cells in the islets by lymphotoxin beta receptor-dependent tertiary lymphoid structure. Immunity 25 (3), 499–509. 10.1016/j.immuni.2006.06.016 16934497

[B41] Lees J. S. Welsh C. E. Celis-Morales C. A. Mackay D. Lewsey J. Gray S. R. (2019). Glomerular filtration rate by differing measures, albuminuria and prediction of cardiovascular disease, mortality and end-stage kidney disease. Nat. Med. 25 (11), 1753–1760. 10.1038/s41591-019-0627-8 31700174 PMC6858876

[B42] Legler D. F. Loetscher M. Roos R. S. Clark-Lewis I. Baggiolini M. Moser B. (1998). B cell-attracting chemokine 1, a human CXC chemokine expressed in lymphoid tissues, selectively attracts B lymphocytes via BLR1/CXCR5. J. Exp. Med. 187 (4), 655–660. 10.1084/jem.187.4.655 9463416 PMC2212150

[B43] Li L. Okusa M. D. (2010). Macrophages, dendritic cells, and kidney ischemia-reperfusion injury. Semin. Nephrol. 30 (3), 268–277. 10.1016/j.semnephrol.2010.03.005 20620671 PMC2904394

[B44] Liu N. Yang X. Yang L. Xu J. Dong R. Li Y. (2021). Establishment of human induced pluripotent stem cell line (SDQLCHi040-A) from a patient with Infantile-onset inflammatory bowel disease carrying a homozygous mutation in IL10RA gene. Stem Cell Res. 56, 102533. 10.1016/j.scr.2021.102533 34530396

[B45] Liu Y. C. Zou X. B. Chai Y. F. Yao Y. M. (2014). Macrophage polarization in inflammatory diseases. Int. J. Biol. Sci. 10 (5), 520–529. 10.7150/ijbs.8879 24910531 PMC4046879

[B46] Livingston M. J. Ding H. F. Huang S. Hill J. A. Yin X. M. Dong Z. (2016). Persistent activation of autophagy in kidney tubular cells promotes renal interstitial fibrosis during unilateral ureteral obstruction. Autophagy 12 (6), 976–998. 10.1080/15548627.2016.1166317 27123926 PMC4922446

[B47] Lu T. T. (2012). Dendritic cells: Novel players in fibrosis and scleroderma. Curr. Rheumatol. Rep. 14 (1), 30–38. 10.1007/s11926-011-0215-5 22006170 PMC5815163

[B48] Lv L. L. Feng Y. Wu M. Wang B. Li Z. L. Zhong X. (2020). Exosomal miRNA-19b-3p of tubular epithelial cells promotes M1 macrophage activation in kidney injury. Cell Death Differ. 27 (1), 210–226. 10.1038/s41418-019-0349-y 31097789 PMC7206053

[B49] Namjou B. Gray-McGuire C. Sestak A. L. Gilkeson G. S. Jacob C. O. Merrill J. T. (2009). Evaluation of C1q genomic region in minority racial groups of lupus. Genes Immun. 10 (5), 517–524. 10.1038/gene.2009.33 19440201 PMC2769492

[B50] Nguyen N. T. Nguyen T. H. Pham T. N. H. Huy N. T. Bay M. V. Pham M. Q. (2020). Autodock Vina adopts more accurate binding poses but Autodock4 forms better binding affinity. J. Chem. Inf. Model 60 (1), 204–211. 10.1021/acs.jcim.9b00778 31887035

[B51] Oh S. H. Sung Y. H. Kim I. Sim C. K. Lee J. H. Baek M. (2019). Novel compound heterozygote mutation in IL10RA in a patient with very early-onset inflammatory bowel disease. Inflamm. Bowel Dis. 25 (3), 498–509. 10.1093/ibd/izy353 30462267

[B52] Ori Y. Bergman M. Bessler H. Zingerman B. Levy-Drummer R. S. Gafter U. (2013). Cytokine secretion and markers of inflammation in relation to acidosis among chronic hemodialysis patients. Blood Purif. 35 (1-3), 181–186. 10.1159/000346689 23463880

[B53] Pei G. Zeng R. Han M. Liao P. Zhou X. Li Y. (2014). Renal interstitial infiltration and tertiary lymphoid organ neogenesis in IgA nephropathy. Clin. J. Am. Soc. Nephrol. 9 (2), 255–264. 10.2215/CJN.01150113 24262509 PMC3913227

[B54] Platnich J. M. Muruve D. A. (2019). NOD-like receptors and inflammasomes: A review of their canonical and non-canonical signaling pathways. Arch. Biochem. Biophys. 670, 4–14. 10.1016/j.abb.2019.02.008 30772258

[B55] Platten M. Youssef S. Hur E. M. Ho P. P. Han M. H. Lanz T. V. (2009). Blocking angiotensin-converting enzyme induces potent regulatory T cells and modulates TH1- and TH17-mediated autoimmunity. Proc. Natl. Acad. Sci. U. S. A. 106 (35), 14948–14953. 10.1073/pnas.0903958106 19706421 PMC2736463

[B56] Quon B. S. Mayer-Hamblett N. Aitken M. L. Smyth A. R. Goss C. H. (2011). Risk factors for chronic kidney disease in adults with cystic fibrosis. Am. J. Respir. Crit. Care Med. 184 (10), 1147–1152. 10.1164/rccm.201105-0932OC 21799076 PMC3262023

[B57] Rabb H. Daniels F. O'Donnell M. HaqM. Saba S. R. Keane W. (2000). Pathophysiological role of T lymphocytes in renal ischemia-reperfusion injury in mice. Am. J. Physiol. Ren. Physiol. 279 (3), F525–F531. 10.1152/ajprenal.2000.279.3.F525 10966932

[B58] Ramnath D. Powell E. E. Scholz G. M. Sweet M. J. (2017). The toll-like receptor 3 pathway in homeostasis, responses to injury and wound repair. Semin. Cell Dev. Biol. 61, 22–30. 10.1016/j.semcdb.2016.08.014 27552920

[B59] Ritchie M. E. Phipson B. Wu D. Hu Y. Law C. W. Shi W. (2015). Limma powers differential expression analyses for RNA-sequencing and microarray studies. Nucleic Acids Res. 43 (7), e47. 10.1093/nar/gkv007 25605792 PMC4402510

[B60] Rogers N. M. Ferenbach D. A. Isenberg J. S. Thomson A. W. Hughes J. (2014). Dendritic cells and macrophages in the kidney: A spectrum of good and evil. Nat. Rev. Nephrol. 10 (11), 625–643. 10.1038/nrneph.2014.170 25266210 PMC4922410

[B61] Ruddle N. H. (2014). Lymphatic vessels and tertiary lymphoid organs. J. Clin. Invest 124 (3), 953–959. 10.1172/JCI71611 24590281 PMC3934190

[B62] Sato Y. Mii A. Hamazaki Y. Fujita H. Nakata H. Masuda K. (2016). Heterogeneous fibroblasts underlie age-dependent tertiary lymphoid tissues in the kidney. JCI Insight 1 (11), e87680. 10.1172/jci.insight.87680 27699223 PMC5033938

[B63] Segerer S. Schlöndorff D. (2008). B cells and tertiary lymphoid organs in renal inflammation. Kidney Int. 73 (5), 533–537. 10.1038/sj.ki.5002734 18094677

[B64] Seleznik G. Seeger H. Bauer J. Fu K. Czerkowicz J. Papandile A. (2016). The lymphotoxin β receptor is a potential therapeutic target in renal inflammation. Kidney Int. 89 (1), 113–126. 10.1038/ki.2015.280 26398497

[B65] Shimizu A. Masuda Y. Kitamura H. Ishizaki M. Sugisaki Y. Yamanaka N. (1996). Apoptosis in progressive crescentic glomerulonephritis. Lab. Invest 74 (5), 941–951. 8642789

[B66] Shouval D. S. Ouahed J. Biswas A. Goettel J. A. Horwitz B. H. Klein C. (2014). Interleukin 10 receptor signaling: Master regulator of intestinal mucosal homeostasis in mice and humans. Adv. Immunol. 122, 177–210. 10.1016/B978-0-12-800267-4.00005-5 24507158 PMC4741283

[B67] Snaedal S. Heimbürger O. Qureshi A. R. Danielsson A. Wikstrom B. Fellstrom B. (2009). Comorbidity and acute clinical events as determinants of C-reactive protein variation in hemodialysis patients: Implications for patient survival. Am. J. Kidney Dis. 53 (6), 1024–1033. 10.1053/j.ajkd.2009.02.008 19394732

[B68] Steines L. Poth H. Herrmann M. Schuster A. Banas B. Bergler T. (2020). B cell activating factor (BAFF) is required for the development of intra-renal tertiary lymphoid organs in experimental kidney transplantation in rats. Int. J. Mol. Sci. 21 (21), 8045. 10.3390/ijms21218045 33126753 PMC7662293

[B69] Steiniger B. Stehling O. Scriba A. Grau V. (2001). Monocytes in the rat: Phenotype and function during acute allograft rejection. Immunol. Rev. 184, 38–44. 10.1034/j.1600-065x.2001.1840104.x 12086320

[B70] Steinmetz O. M. Lange-Hüsken F. Turner J. E. Vernauer A. Helmchen U. Stahl R. A. K. (2007). Rituximab removes intrarenal B cell clusters in patients with renal vascular allograft rejection. Transplantation 84 (7), 842–850. 10.1097/01.tp.0000282786.58754.2b 17984836

[B71] Stenvinkel P. Ketteler M. Johnson R. J. Lindholm B. Pecoits-Filho R. Riella M. (2005). IL-10, IL-6, and TNF-alpha: Central factors in the altered cytokine network of uremia--the good, the bad, and the ugly. Kidney Int. 67 (4), 1216–1233. 10.1111/j.1523-1755.2005.00200.x 15780075

[B72] Subramanian A. Tamayo P. Mootha V. K. Mukherjee S. Ebert B. L. Gillette M. A. (2005). Gene set enrichment analysis: A knowledge-based approach for interpreting genome-wide expression profiles. Proc. Natl. Acad. Sci. U. S. A. 102 (43), 15545–15550. 10.1073/pnas.0506580102 16199517 PMC1239896

[B73] Szklarczyk D. Santos A. von Mering C. Jensen L. J. Bork P. Kuhn M. (2016). Stitch 5: Augmenting protein-chemical interaction networks with tissue and affinity data. Nucleic Acids Res. 44 (D1), D380–D384. 10.1093/nar/gkv1277 26590256 PMC4702904

[B74] Taminau J. Meganck S. Lazar C. Steenhoff D. Coletta A. Molter C. (2012). Unlocking the potential of publicly available microarray data using inSilicoDb and inSilicoMerging R/Bioconductor packages. BMC Bioinforma. 13, 335. 10.1186/1471-2105-13-335 PMC3568420 23259851

[B75] Toyama S. Yamashita T. Saigusa R. Miura S. Nakamura K. Hirabayashi M. (2020). Decreased serum cathepsin S levels in patients with systemic sclerosis-associated interstitial lung disease. J. Dermatol 47 (9), 1027–1032. 10.1111/1346-8138.15458 32515028

[B76] Wen Y. Yan H. R. Wang B. Liu B. C. (2021). Macrophage heterogeneity in kidney injury and fibrosis. Front. Immunol. 12, 681748. 10.3389/fimmu.2021.681748 34093584 PMC8173188

[B77] White S. Lin L. Hu K. (2020). NF-κB and tPA signaling in kidney and other diseases. Cells 9 (6), 1348. 10.3390/cells9061348 32485860 PMC7348801

[B78] Yao X. Cheng F. Yu W. Rao T. Li W. Zhao S. (2019). Cathepsin S regulates renal fibrosis in mouse models of mild and severe hydronephrosis. Mol. Med. Rep. 20 (1), 141–150. 10.3892/mmr.2019.10230 31115520 PMC6580002

[B79] Zhao S. Y. Liao L. X. Tu P. F. Li W. W. Zeng K. W. (2019). Icariin inhibits AGE-induced injury in PC12 cells by directly targeting apoptosis regulator bax. Oxid. Med. Cell Longev. 2019, 7940808. 10.1155/2019/7940808 31178973 PMC6501163

[B80] Zimmermann J. Herrlinger S. Pruy A. Metzger T. Wanner C. (1999). Inflammation enhances cardiovascular risk and mortality in hemodialysis patients. Kidney Int. 55 (2), 648–658. 10.1046/j.1523-1755.1999.00273.x 9987089

[B81] Zoccali C. Tripepi G. Mallamaci F. (2006). Dissecting inflammation in ESRD: Do cytokines and C-reactive protein have a complementary prognostic value for mortality in dialysis patients? J. Am. Soc. Nephrol. 17 (3), S169–S173. 10.1681/ASN.2006080910 17130257

