# Supplementary material for: Identifying effective diagnostic biomarkers and immune infiltration features in chronic kidney disease by bioinformatics and validation
Source: Front Pharmacol. 2022 Dec 30;13:1069810. doi: 10.3389/fphar.2022.1069810 (PMC9838551; doi:10.3389/fphar.2022.1069810)
Supplement: Supplementary file 2 [file DataSheet4.PDF]

编 号：KYXM-202208-006

安徽医科大学附属巢湖医院医学伦理委员会
课题论证报告
（正　本）

课题负责人：庄星星

课题名称：FTO 介导 HMGB1 m6A 甲基化修饰调控肾小球系膜细胞增殖和凋亡的机制研究

承担单位：安徽医科大学附属巢湖医院

一、伦理委员会对该课题方案进行了论证，并特别对以下三方面进行了认真讨论：
1、研究对象的权利与利益；
2、确保取得知情同意的措施；
3、存在的危险与可能的受益。

二、同意实施该课题方案。实施过程中请使用经论证的知情同意书、问卷、说明信等材料。

三、课题方案如需修改，须事先经伦理委员会论证方可实施，修改内容及其原因需详细备案。

四、实施过程中如出现任何不良反应需立即向伦理委员会做出书面报告。

委员签字：[illegible]

[illegible]

[illegible]

（盖 章）

安徽医科大学附属巢湖医院 医学伦理委员会

2022年 8月22日
