# Supplementary material for: Identifying effective diagnostic biomarkers and immune infiltration features in chronic kidney disease by bioinformatics and validation
Source: Front Pharmacol. 2022 Dec 30;13:1069810. doi: 10.3389/fphar.2022.1069810 (PMC9838551; doi:10.3389/fphar.2022.1069810)
Supplement: Supplementary file 5 [file DataSheet5.ZIP › Additional file/original image of immunohistochemistry.docx]

https://www.jianguoyun.com/p/Df4BXKMQh4uGCxj1juAEIAA
